# Winning entry of the creative competition

**DOI:** 10.1007/s15010-024-02299-6

**Published:** 2024-05-24

**Authors:** Ulrich Kaiser

**Affiliations:** Clinic and Polyclinic for Internal Medicine III, University Hospital Regensburg, Regensburg, Germany

## Image description

Award of the 2023 creative competition. In the absence of clinical symptoms of a urinary tract infection, antibiotic therapy is not necessary if bacteriuria is detected (asymptomatic bacteriuria), with a few exceptions.
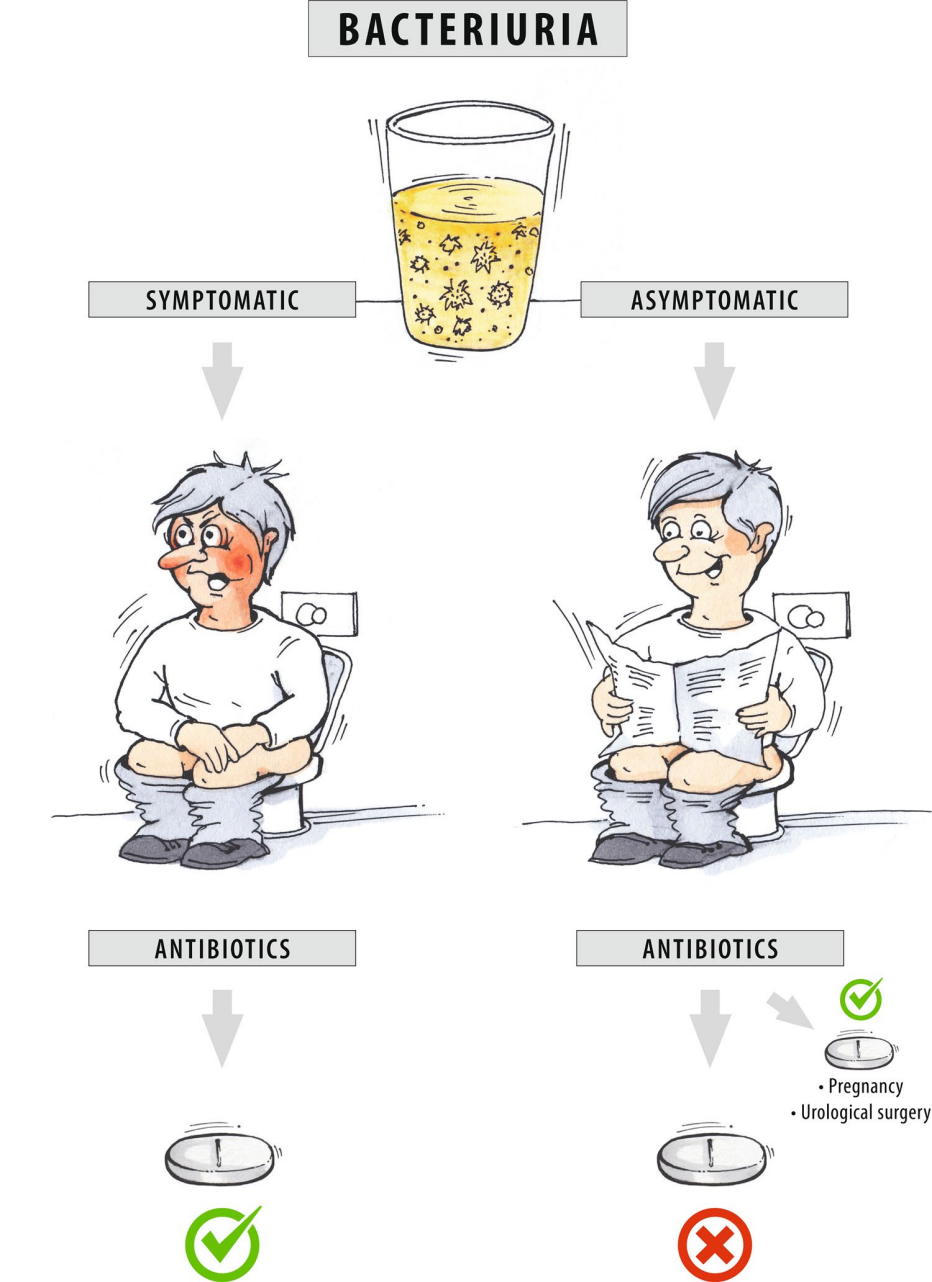


## Data Availability

No datasets were generated or analysed during the current study.

